# Low level of Lck kinase in Th2 cells limits expression of CD4 co-receptor and S73 phosphorylation of transcription factor c-Jun

**DOI:** 10.1038/s41598-017-02553-y

**Published:** 2017-05-24

**Authors:** Yury V. Shebzukhov, Silke Stanislawiak, Taisiya R. Bezhaeva, Sergei A. Nedospasov, Dmitry V. Kuprash

**Affiliations:** 1 German Rheumatism Research Center, a Leibniz Institute, Berlin, Germany; 2 Engelhardt Institute of Molecular Biology, Russian Academy of Sciences, Moscow, Russia; 3 Lomonosov Moscow State University, Moscow, Russia

## Abstract

The Src-family tyrosine kinase Lck is an enzyme associated with the CD4 and CD8 co-receptors and promoting signaling through the T cell receptor (TCR) complex. The levels of Lck expression and activity change during the development and differentiation of T cells. Here we show that Lck expression is higher in Th1 cells as compared to Th2 cells. Ectopic overexpression of Lck in Th2 cells results in increased expression of CD4 co-receptor and enhanced S73 phosphorylation of transcription factor c-Jun. Our findings indicate that TCR-mediated signaling in Th2 cells may be directly attenuated by Lck protein expression level.

## Introduction

Th1 and Th2 cells were discovered in the late 1980s as T helper lymphocyte subsets producing different sets of cytokines and promoting cellular (Th1 cells) and humoral (Th2 cells) immune responses (reviewed in ref. 1). Quantitative differences in T cell receptor (TCR)-induced signaling between Th1 and Th2 cells were reported soon after their discovery, and included lower Ca^2+^ flux and lower generation of inositol phosphates in Th2 compared to Th1 cells^2, 3^. Upon antigen stimulation, the proximal TCR signaling complex containing protein tyrosine kinases Zap70 and Fyn and the TCR signaling component CD3ζ/TCR-ζ was less activated in Th2 compared to Th1 cells, as reflected by less efficient complex formation and reduced phosphorylation^4–7^. The differences in morphology and function of immunological synapses (IS) were also evident in these T cell subsets, with less efficient CD4-TCR clustering and recruitment of TCR components in Th2 as compared to Th1 cells^8–10^.

Further differences between Th1 and Th2 cells were reported downstream of the proximal TCR signaling complex. In particular, lower activation of the c-Jun N-terminal kinases (JNK) and decreased nuclear localization of NFATc2 and RelA transcription factors in Th2 cells were observed^11–13^. We have also reported lower level of nuclear localisation of the JNK substrate transcription factor c-Jun in Th2 as compared to Th1 cells^14^.

Expression of several proteins involved in the proximal TCR signaling is downregulated in Th2 cells. First, reduced surface expression of the CD4 co-receptor on Th2 lymphocytes contributes to the suboptimal proximal TCR signaling in these cells^7^. Second, the level of the TCR-associated protein tyrosine kinase Fyn is lower in Th2 as compared to Th1 cells^6^. Additionally, downstream of the proximal TCR complex and the LAT signalosome, several components of kinase cascades are attenuated. In particular, the level of small GTPase RAC2 that activates MAP3Ks MEKK1 and MLK3, is lower in Th2 cells^15^, while phosphatase DUSP16/MKP-7 limiting the activity of JNK and ERK cascades is expressed at much higher level in Th2 than in Th1 cells^16, 17^.

Here we show that tyrosine kinase Lck that is associated with CD4 and CD8 co-receptors is also expressed at a lower level in Th2 as compared to Th1 cells. Ectopic Lck overexpression in Th2 cells increased expression of CD4 co-receptor and augmented S73 phosphorylation of transcription factor c-Jun.

## Results

### Lck expression in Th2 cells as compared to Th1 cells is reduced at both protein and mRNA levels

**Figure 1:**
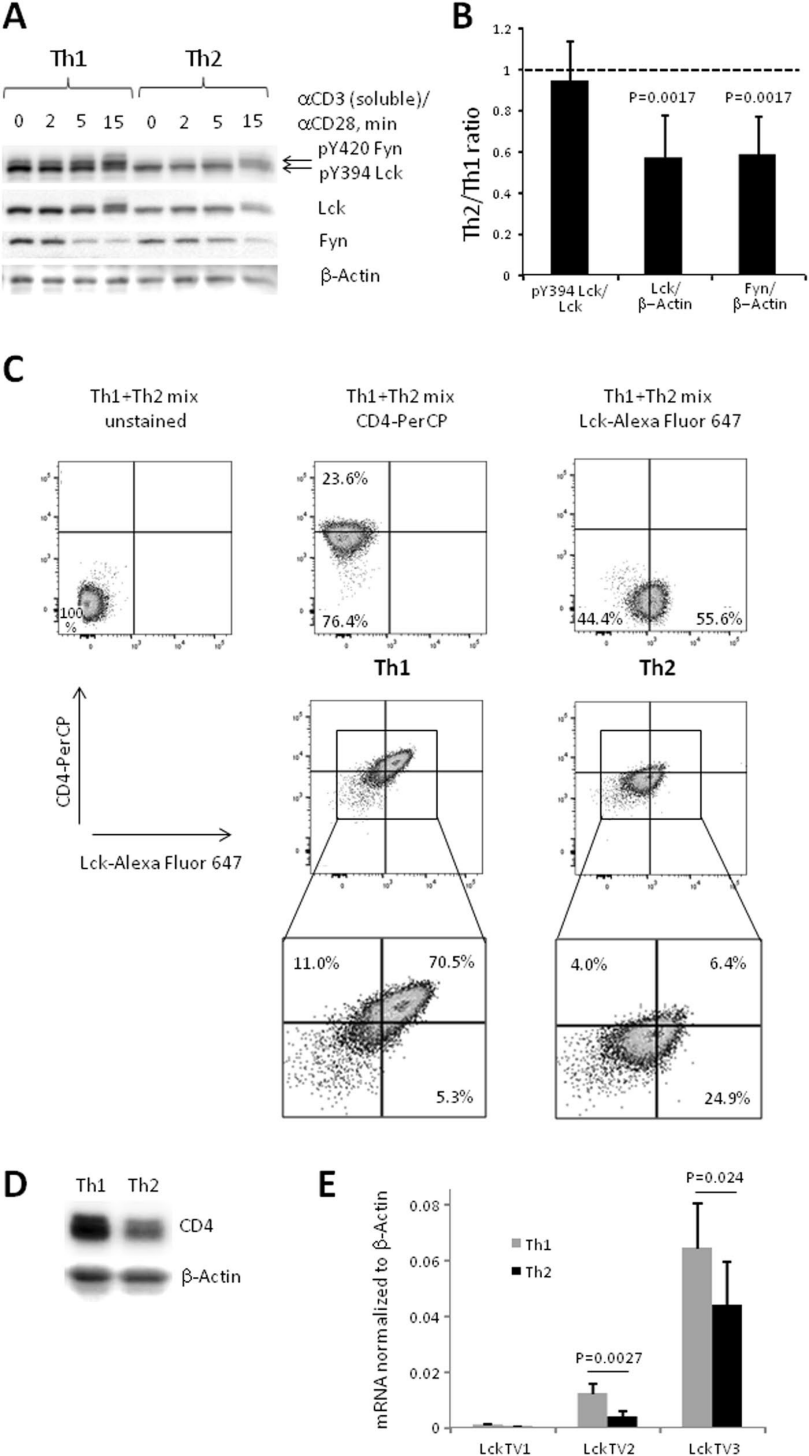
Reduced Lck and CD4 expression in mouse Th2 cells. Naive CD4^+^ T cells were polarized under Th1 and Th2 conditions for 5 days, rested overnight without APCs, antibodies and cytokines and re-stimulated with anti-CD3 (10 µg/ml) and anti-CD28 (2 µg/ml) antibodies. (**A**,**D**) Western blotting analysis of cytoplasmic/cell membrane fraction (**A**) or total cell lysate (**D**). Results of a representative experiment of four experiments are shown. (**B**) Densitometry analysis of Western blot images of resting Th1 and Th2 cells. Average and standard deviation of three (pY394Lck/total Lck ratio) and seven (total Lck/β-Actin and total Fyn/β-Actin ratios) experiments are shown. Mann Whitney U test was used to perform statistical comparisons (only for total Lck/β-Actin and total Fyn/β-Actin ratios). (**C**) Flow cytometry analysis of Th1 and Th2 cells. Results of a representative experiment (of four experiments) are shown. (**E**) Lck mRNA expression in Th1 and Th2 cells. Lck V1 and Lck V2–transcripts from the proximal promoter, Lck V3–transcript from the distal promoter. Average and standard deviation of eight independent experiments are shown. Student's *t*-test was used to perform statistical comparisons.

We asked whether a weaker TCR-mediated response in Th2-polarized T cells relative to Th1 cells may be due to reduced expression of tyrosine kinases that initiate the TCR signaling. In order to test this hypothesis, we assessed protein levels of the Src-family tyrosine kinase Lck in these T cell subsets using Western blotting (Fig. 1A) and performed comparative densitometry analysis for resting Th1 and Th2 cells (Fig. 1B). We found that both the total protein expression level and the amount of the phosphorylated Lck were lower in Th2 cells as compared to Th1 cells (Fig. 1A,B). However, relative Lck activating phosphorylation measured as a ratio of pY394 Lck to total Lck was comparable between resting Th1 and Th2 cells (Fig. 1B). Both naive CD4^+^ cells and Th0 cells differentiated under neutral conditions demonstrated total Lck protein level similar to that observed in Th1 cells (Supplementary Fig. S1). However, the level of phosphorylated Lck was lower in naive CD4^+^ T cells as compared to differentiated T cell subsets (Supplementary Fig. S1).

In Th2 cells we could also confirm the previously reported reduced level of Fyn, another Src-family member involved in TCR signaling (Fig. 1A,B)^4, 6^. We observed a lower expression level of Lck in Th2 as compared to Th1 cells by intracellular staining and flow cytometry (Fig. 1C and Supplementary Fig. S2). Notably, surface CD4 level assessed by flow cytometry correlated with Lck expression and was lower in Th2 as compared to Th1 cells in accord with the results reported by Itoh *et al*.^7^ (Fig. 1C and Supplementary Fig. S2). Total CD4 protein level assessed by Western blotting was also lower in Th2 compared to Th1 cells (Fig. 1D), however, we did not observe any difference in CD4 mRNA levels between these cells (Supplementary Fig. S3).

*Lck* gene has two promoters: the proximal promoter driving Lck expression both in double negative (DN) and in double positive (DP) thymocytes and the distal promoter that starts to operate only in DP thymocytes and remains active at all subsequent stages of the T cell development and differentiation^18, 19^. We designed PCR primers to selectively detect Lck mRNAs originating from the proximal (transcript variants 1 (TV1), NM_001162432 and 2 (TV2), NM_010693) and the distal (transcript variant 3 (TV3), NM_001162433) promoters and used Q-RT-PCR to quantify these transcripts in mouse T cells polarized under Th1 and Th2 conditions. TV3 was the dominant *Lck* mRNA isoform in all cells tested while TV1 level was barely detectable. Both TV3 and TV2 demonstrated a moderate, but statistically significant increase in cells polarized under Th1 conditions as compared to Th2 cells (Fig. 1E). In naive CD4^+^ and Th0 cells we found intermediate mRNA expression profiles of TV2 and TV3. In particular, TV2 was expressed at comparable levels in naive CD4^+^, Th0 and Th1 cells, while TV3 was expressed at comparable levels in naive CD4^+^, Th0 and Th2 cells (Supplementary Fig. S4).

The analysis of independent microarray data sets deposited to Gene Expression Omnibus (GEO) repository have also indicated that Lck mRNA expression was moderately, but consistently higher in Th1 as compared to Th2 cells (Supplementary Fig. S5).

Since Lck transcripts 2 and 3 encode the same protein (Lck isoform b), we concluded that the difference in Lck protein expression between Th1 and Th2 cells is due, at least partly, to the difference in Lck mRNA levels.

### Lck modulates protein expression of CD4 co-receptor in Th2 cells

**Figure 2:**
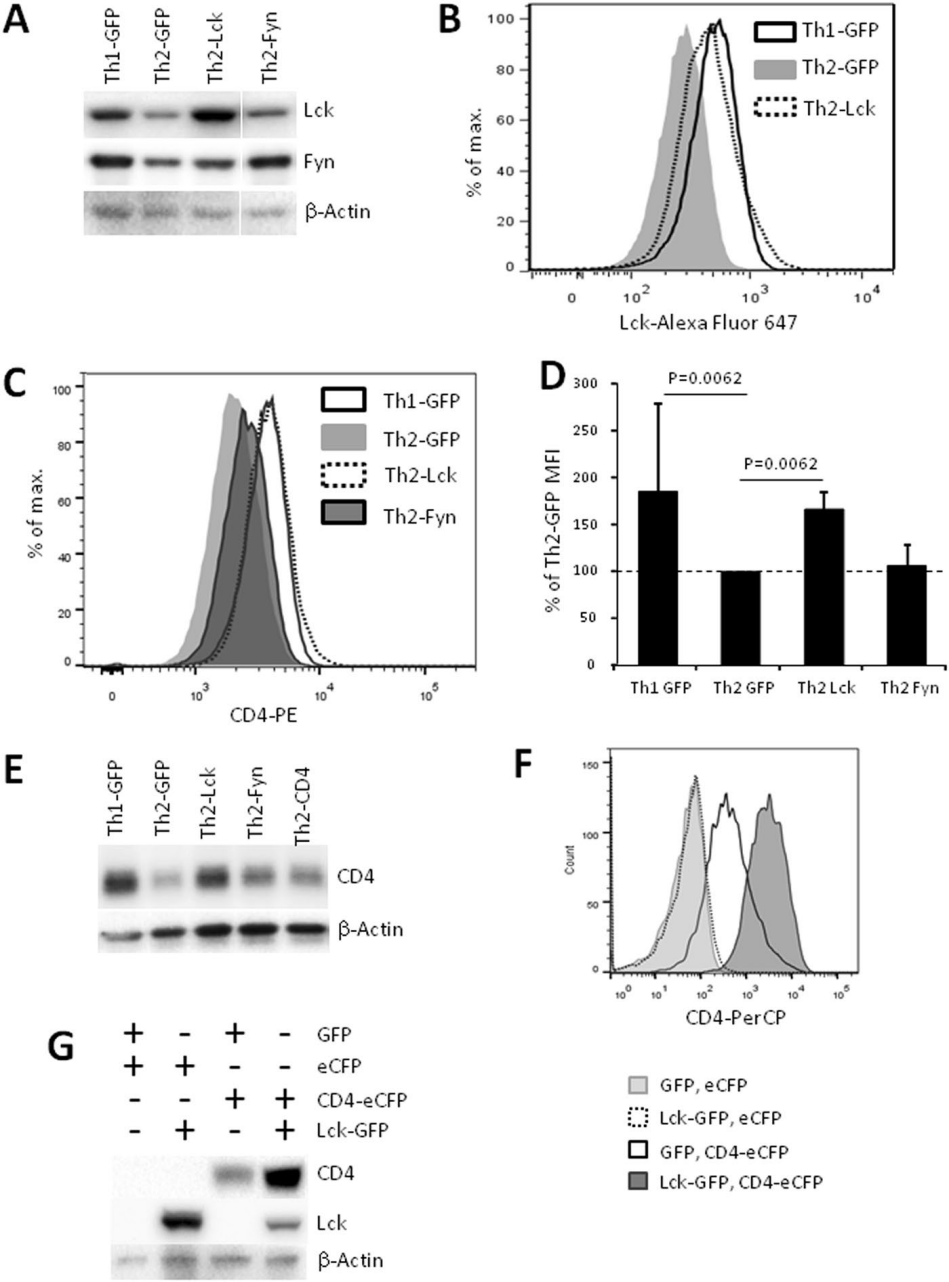
Lck positively regulates CD4 expression. Naive CD4^+^ cells were transduced with control (pMSCV-IRES-GFP), Lck (pMSCV-LCK-IRES-GFP), Fyn (pMSCV-FYN-IRES-GFP) or CD4 (pMSCV-CD4-IRES-GFP) encoding retroviruses and polarized under Th2 or Th1 (control vector only) conditions. 5 days after initiation of cell cultures GFP^+^ cells were isolated and analyzed by Western blotting and flow cytometry. HEK293T cells were transfected by control (pMSCV-IRES-GFP, pMSCV-IRES-eCFP), Lck (pMSCV-LCK-IRES-GFP) or CD4 (pMSCV-CD4-IRES-eCFP) encoding vectors. (**A**,**E**) Western blot of isolated GFP^+^ cells. Results of a representative experiment of two experiments are shown. (**A**) Cropped blots are shown. Complete blots are shown in Supplementary Fig. S6. Staining was performed using the same blots as in Fig. 3A. Correspondingly, the same β-Actin staining is shown there as loading control. (**B**,**C**) Flow cytometry analysis of isolated GFP^+^ cells. Results of a representative experiment ((**B**) of four experiments, (**C**) of five experiments). (**D**) Mean PE fluorescence of GFP^+^ cells normalized to that of Th2 cells transduced with control retrovirus. Average and standard deviation of five experiments are shown. Mann Whitney U test was used to perform statistical comparisons. (**F**,**G**) Flow cytometry (**F**) and Western blot (**G**) analysis of GFP^+^eCFP^+^ transfected HEK293T cells. Results of a representative experiment of two experiments are shown.

To further address specific functional consequences of the relative Lck deficit in Th2 cells, we used retroviral transduction to overexpress Lck and Fyn and analyzed transduced cells by flow cytometry and Western blot. We found that Lck and Fyn-transduced Th2 cells express Lck and Fyn at levels comparable with those in Th1 cells transduced with control vector (Fig. 2A,B and Supplementary Fig. S6).

Importantly, the surface expression of the CD4 co-receptor in Lck-transduced Th2 cells was elevated to the level similar to that observed in Th1 cells, while overexpression of Fyn did not have such effect (Fig. 2C,D, Supplementary Fig. S7A,B and Supplementary Table S1). The total level of CD4 protein in Th2 cells as analyzed by Western blot was also increased upon overexpression of Lck but not of Fyn (Fig. 2E). Surprisingly, transduction of Th2 cells with retroviruses encoding CD4 did not result in an increase of CD4 surface or total protein expression (Fig. 2E, Supplementary Fig. S7A,B and Supplementary Table S1). However, human HEK293T cells transfected with the vector used for generation of CD4-encoding retrovirus expressed CD4, as assessed by Western blot and flow cytometry analyses (Supplementary Fig. S7C,D).

Positive regulation of CD4 by Lck was described before^20–24^ and we hypothesized that it is the level of Lck expression that limits CD4 expression in Th2 cells. To analyze effect of Lck on CD4 expression independently of other TCR components and T-cell specific surface molecules we co-transfected human HEK293T cells with eCFP-labeled vector encoding CD4 and with GFP-labeled vector encoding Lck. We observed a significant increase in both surface and total CD4 expression in double positive eCFP^+^GFP^+^ cells expressing both Lck and CD4 as compared to cells expressing CD4 alone (Fig. 2F,G).

### Lck and Fyn increase serine phosphorylation in position 73 of transcription factor c-Jun in Th2 cells

**Figure 3:**
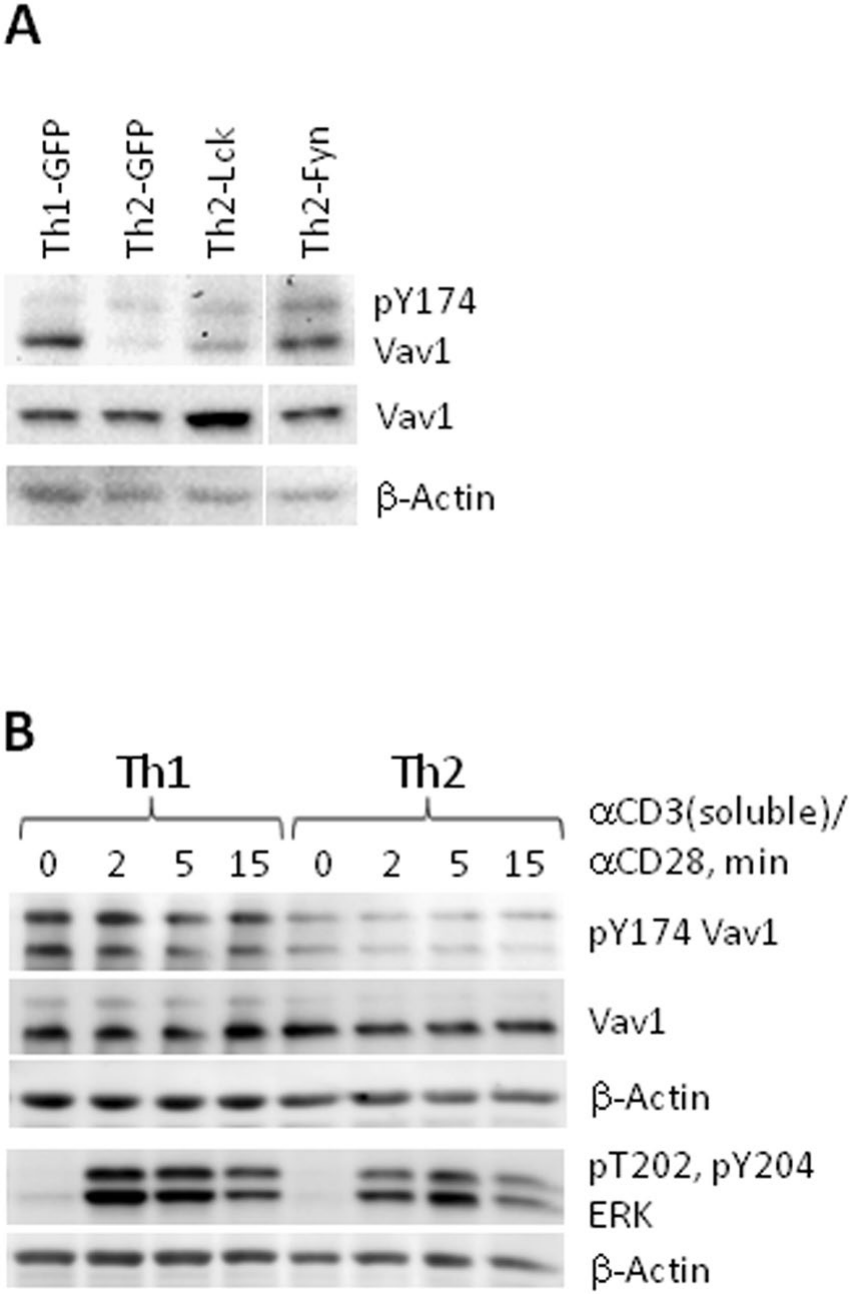
Effect of Lck and Fyn ectopic expression in Th2 cells on Y174 VAV1 phosphorylation. Naive CD4^+^ cells were transduced (A only) with control, Lck and Fyn encoding retroviruses and polarized (**A**,**B**) under Th2 or Th1 (control vector only) conditions. 5 days after initiation of cell cultures GFP^+^ (**A**) or total (**B**) cells were isolated, rested overnight without APCs, antibodies and cytokines and re-stimulated (B only) with anti-CD3 (10 µg/ml) and anti-CD28 (2 µg/ml) antibodies. Western blotting of total cell lysate (**A**) or of cytoplasmic/cell membrane fraction (**B**). (**A**) Cropped blots are shown. Complete blots are shown in Supplementary Fig. S9. Staining was performed using the same blots as in Fig. 2A. Correspondingly, the same β-Actin staining is shown there as loading control. Results of a representative experiment of two experiments are shown.

We next analyzed phosphorylation levels of the TCR-associated signaling molecules. There was no significant increase in phosphorylation of CD3-ζ component of TCR complex or of phospholipase C-γ (PLCγ) either in Lck- or in Fyn-transduced Th2 cells (Supplementary Fig. S8A,B). However, phosphorylation of guanine nucleotide exchange factor (GEF) VAV1 linking LAT signalosome to small GTPases RAC/CDC42 of the Rho family was elevated in Fyn-transduced Th2 cells (Fig. 3A and Supplementary Fig. S9). This result was in accord with the previous report by Michel *et al*.^25^ that Fyn is required for VAV phosphorylation. We also observed that endogenous phosphorylation level of VAV in both resting and activated Th1 cells was higher as compared to Th2 cells (Fig. 3B).

Overexpression of Lck, but not of Fyn, resulted in moderate increase of TCR-induced phosphorylation of mitogen activated protein kinases ERK1/2 when stimulated with soluble anti-CD3 and anti-CD28 antibodies (Supplementary Fig. S10), but this effect was not statistically significant. Overexpression of neither Lck nor Fyn in Th2 cells affected phosphorylation of the stress-activated kinases JNK1/2 (Supplementary Fig. S11).

**Figure 4:**
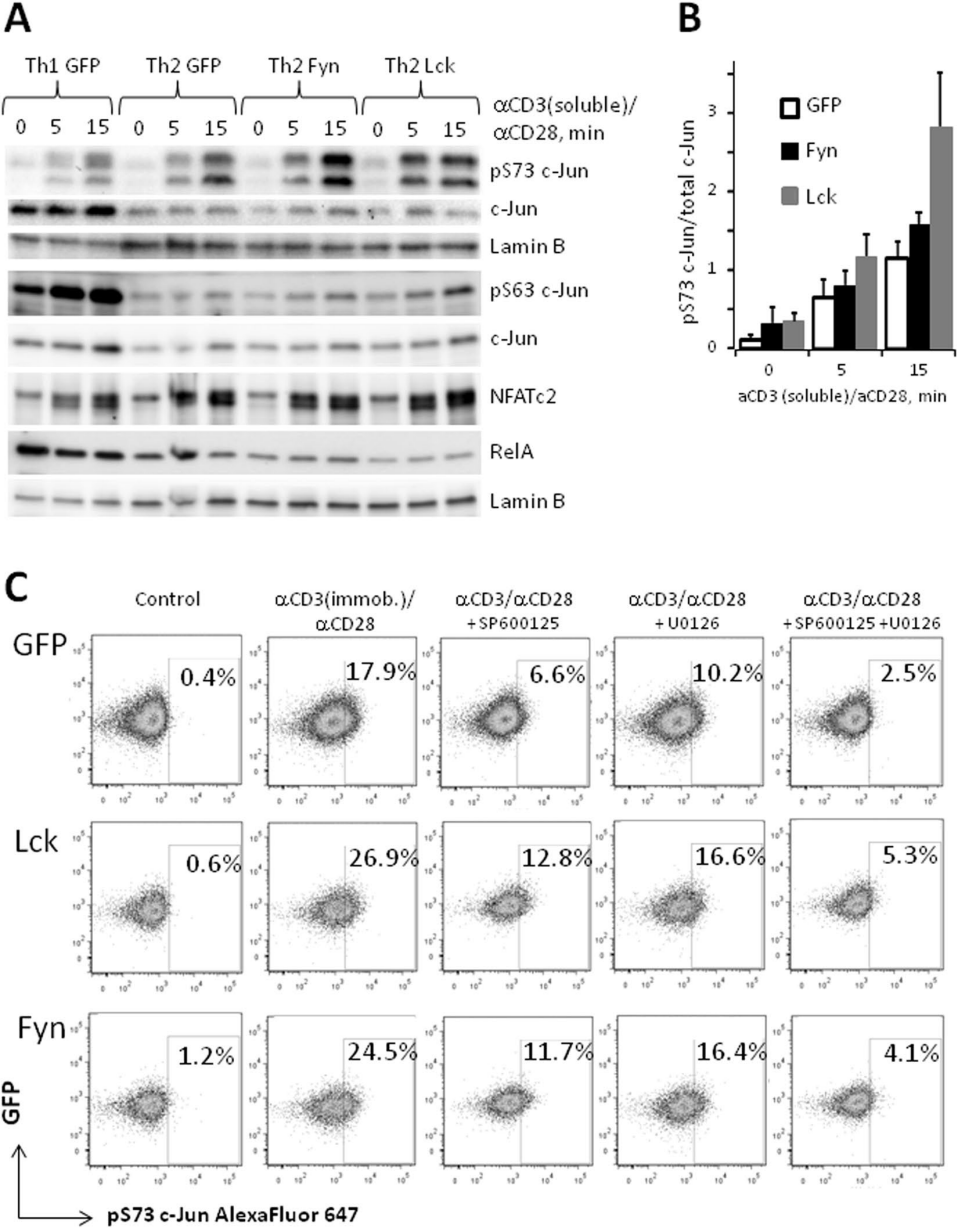
Ectopic expression of Lck and Fyn in Th2 cells increases S73 phosphorylation of c-Jun. Naive CD4^+^ cells were transduced and polarized as described in the legend to Fig. 2. 5 days after initiation of cell culture GFP^+^ cells were isolated, rested overnight without APCs, antibodies and cytokines and re-stimulated with anti-CD3 and anti-CD28 antibodies. (**A**) Western blot of nuclear fraction of cell re-stimulated with soluble anti-CD3 and anti-CD28 antibodies. Results of a representative experiment of three experiments are shown. (**B**) Densitometry analysis of Western blot images of pS73 and total c-Jun from (**A**). Signals obtained from pS73 c-Jun blots were divided to signals of total c-Jun blots and normalized to the mean signals ratio for each experiment. Average and standard deviation of three experiments are shown. Densitometry data used for generation of histogram are shown in Supplementary Table S2. (**C**) Flow cytometry analysis of GFP^+^ cells re-stimulated with immobilized anti-CD3 and soluble anti-CD28 antibodies. Inhibitors of JNK (SP600125, 50 µM) and ERK (U0126, 10 µM) cascades are added 1 hour before cells re-stimulation. Results of a representative experiment of three experiments are shown.

Analysis of nuclear concentrations of the key TCR-activated transcription factors NFATc2, RelA/p65 and c-Jun (AP-1 family) did not reveal any significant effects of Lck or Fyn overexpression. However, activating phosphorylation of c-Jun at Ser73 in response to TCR stimulation was moderately increased both in Lck- and in Fyn-transformed Th2 cells (Fig. 4). Somewhat surprisingly, activating phosphorylation of c-Jun at Ser63 was not affected by ectopic overexpression of Lck or Fyn (Fig. 4A). As expected, control Th1 cells demonstrated higher levels of c-Jun expression and phosphorylation; however pS73 phosphorylation was observed in these cells only at a later time point after stimulation (Fig. 4A and Supplementary Fig. S12).

Inhibitors of ERK (U0126) and JNK (SP600125) pathways both decreased S73 phosphorylation of c-Jun, with higher residual activation in Lck- and Fyn-transduced Th2 cells and with strongest inhibition observed upon combined pre-treatment of cells with both compounds (Fig. 4C). Thus, ectopic overexpression of Lck and Fyn in Th2 cells increased the activities of both JNK and ERK pathways which acted in concert, however, significant increase of activating phosphorylation of both MAP kinases was not observed and the upstream signaling mechanisms remained obscure.

### Lck overexpression in Th2 cells has limited effect on cytokine gene expression

To investigate the effect of Lck overexpression on the cytokine profile of Th2 cells, we performed transcription analysis for the genes that encode pro-inflammatory (IL2, TNF) and anti-inflammatory (IL4, IL10) cytokines. All cells demonstrated similar fast kinetics of cytokine mRNA expression, regardless of the Lck or Fyn retroviral overexpression, with the only exception of IL2 mRNA in Lck-transduced cells. In the latter case, IL2 mRNA level was increased 1 hour after TCR activation and continued to accumulate at 3 hour time point (Supplementary Fig. S13). IL2 mRNA level in Fyn-transduced cells was also increased as compared to control, albeit to a lesser extent, and did not grow further at 3 hours. However, we did not observe differences in IL2 protein production between Th2 cells transduced by Lck- or Fyn- encoding and control retroviruses (Supplementary Fig. S14), probably, due to IL2 consumption by activated T cells. Overall, we concluded that ectopic overexpression of Lck and Fyn in Th2 cells has limited effect on cytokine expression.

## Discussion

The differences in TCR-mediated signaling between Th1 and Th2 cells are known for more than 25 years, but underlying molecular mechanisms are still not completely understood. One of the open questions is related to the role of the Lck and its interplay with TCR subunits and associated signaling molecules. The major binding partner of Lck in T helper lymphocytes is CD4 co-receptor^26^ and several independent research groups have shown that Lck prevents CD4 endocytosis and proteolytic degradation^27–29^ and positively regulates surface CD4 level^20–24^.

Lck also interacts with CD28 co-receptor^30, 31^ and transmits activating signal after CD28 stimulation^22, 32, 33^. Our results concerning high Lck expression in Th1 cells and positive effects of Lck overexpression in Th2 cells on CD4 level and phosphorylation status of transcription factor c-Jun correlate well with higher TCR-mediated signal in Th1 as compared to Th2 cells.

However, regardless of the lower Lck expression and activity in Th2 subset, Lck is critically required for the proper differentiation and function of these cells^20, 21, 34–36^, particularly, for the expression of Th2 master regulator GATA3 and the lineage specific cytokine IL4^20, 21, 34, 36^.

In our experiments Lck expression level was critical for the CD4 protein expression, since CD4 mRNA levels were similar in Th1 and Th2 cells and retroviral transduction of Th2 cells with CD4-encoding vector was unable to increase the CD4 level. Our results appear to be at variance with data from Itoh *et al*.^7^ who observed similar Lck levels in Th1 and Th2 cells and were able to increase CD4 expression in Th2 cells to its level in Th1 subset by retroviral transduction. According to Itoh *et al*., overexpression of CD4 in Th2 cells could rescue proximal TCR signaling, particularly, CD3-ζ and ZAP70 phosphorylation and Ca2^+^ response^7^. However, the downstream signaling events in Th2 cells after ectopic CD4 overexpression were not analysed.

In our experiments the Lck protein expression in Th1 and Th2 cells correlated with Lck mRNA levels. Differences in Lck expression in Th1 and Th2 cells may result from heterogeneity of the initial polyclonal population of naive CD4^+^ T cells polarized under Th1 and Th2 conditions. It was shown earlier that stronger TCR signaling promotes development of Th1 cells, while signals of intermediate strength are favourable for development of the Th2 subset^37^. In our experiments we used wild type mice and applied polyclonal stimulation by anti-CD3 and anti-CD28 antibodies. Under these circumstances one may expect that naive T cells with initially higher Lck expression (and correspondingly stronger TCR-mediated signaling) will be more effectively proliferating upon stimulation under Th1 polarizing conditions while the cells with lower Lck levels will be more readily differentiating in the Th2 direction. One possible reason why Itoh *et al*. did not observe the difference in Lck expression between Th1 and Th2 cells may be due to the use of homogenous naive T cells from TCR-transgenic 5 C.C7 mice on RAG-2^−^ background (lacking endogenous TCR molecules) stimulated with specific antigenic peptide^7^.

In contrast to Lck, ectopic expression of Fyn did not modulate CD4 levels in Th2 cells (Fig. 2C–E and Supplementary Fig. S7A,B), but increased phosphorylation of GEF VAV1 (Fig. 3A) which is involved in TCR-mediated signaling downstream signalosome complex assembled around linker for activation of T cells (LAT)^38^.

Overexpression of Lck and Fyn resulted in increased phosphorylation of c-Jun (Fig. 4A–C and Supplementary Table S2), but not of JNK (Supplementary Fig. S11). While c-Jun is the classical substrate of JNK^39^, ERK can also directly phosphorylate it^40^ and we observed a moderate increase of ERK phosphorylation in Lck-transduced Th2 cells (Supplementary Fig. S10). Since inhibitors of both ERK and JNK cascades could decrease TCR-induced c-Jun phosphorylation (Fig. 4C), we concluded that ectopic expression of Lck and Fyn in Th2 cells increased the activity of both ERK and JNK pathways, however, the exact signaling mechanisms remain to be defined.

Overall, our study demonstrates that low level of Lck in Th2 cells limits CD4 expression and activation of transcription factor c-Jun. This knowledge will be useful for future dissection of signaling mechanisms in T cells under homeostatic and pathological conditions.

## Materials and methods

### Laboratory animals

C57BL6 mice were purchased from Charles River Laboratories and maintained under specific pathogen-free conditions. All animal experiments were performed in accordance with institutional, state, and federal guidelines (State Office for Public Health and Social Affairs/Landesamt für Gesundheit und Soziales - LAGeSo, Berlin, Germany). All animal-related protocols were approved by LAGeSo (licence #T0385/10).

### Cell culture

Total cells were isolated from spleen, mesenterial, popliteal and auxiliary lymph nodes and CD4^+^CD62L^+^CD44^−^CD25^−^ naïve T cells were purified by magnetic-activated cell sorting (MACS) technology (Miltenyi Biotec, Bergisch Gladbach, Germany)^41^. Th1 and Th2 cells were polarized as described^14^. Briefly, for differentiation of the Th1 subset, naive CD4^+^ T cells were incubated with 10 ng/ml of recombinant IL-12 in presence of 10 μg/ml anti-IL-4 antibodies. For differentiation of Th2 subset, cells were incubated with 10 ng/ml of recombinant IL-4 in presence of 10 μg/ml anti-IFN-γ and anti-IL-12 antibodies. Naive CD4^+^ T cells were stimulated with 4 μg/ml of immobilized anti-CD3 and with 1 μg/ml of soluble anti-CD28 antibodies. Irradiated CD4^−^ cells were used as antigen presenting cells (APC) and added in the ratio 5/1. At day 3 cell cultures were supplemented with 10 ng/ml of recombinant IL-2. Polarized T cells were isolated at day 5 by centrifugation in Ficoll gradient, rested overnight in complete medium without APC, cytokines or antibodies and re-stimulated with 10 µg/ml of anti-CD3 and 2 µg/ml of anti-CD28 soluble antibodies. Efficiency of Th1 and Th2 differentiation was monitored by expression of corresponding lineage-specific cytokines (IFNγ and IL-4) and transcription factors (T-bet and GATA3) (Supplementary Fig. S15). All antibodies used for cell differentiation, activation and analysis are listed in Supplementary Table S3.

### Retroviral transduction

Plasmids encoding mouse Lck (Ref. Seq. BC011474, cat. #MC203794) and Fyn (Ref. Seq. BC032149, cat. #MC204024) were purchased from OriGene Inc., Rockville, MD. Lck and Fyn coding sequences were excised from pCMV6-Kan/Neo plasmid and re-cloned to pMSCV-IRES-GFP vector (https://www.addgene.org/20672/). CD4 cDNA was amplified using total Th1 RNA and oligo-dT_18_ primer for reverse transcription and specific primers for PCR (Supplementary Table S4). CD4 cDNA was cloned to pMSCV-IRES-GFP and pMSCV-IRES-eCFP vectors. pMSCV-IRES-eCFP vector was generated by replacement of GFP coding sequence by eCFP coding sequence excised from pECFP-N1 plasmid (https://www.addgene.org/vector-database/2446/). T cells were retrovirally transduced as described^7^ with minor modifications. Briefly, packaging cells HEK293T were transfected with appropriate vectors and auxiliary plasmids pECO (encoding viral env protein) and pCGP (encoding the viral gag and pol proteins) using Ca^2+^-phosphate method. The medium containing viral particles (viral stock) was collected 24 and 48 hours after transfection and supplemented with the HEPES buffer to final concentration 10 mM. Murine naive CD4^+^ T cells were cultured for 24 h under Th1 or Th2 polarizing conditions as described above. Culture medium from T cells was replaced by viral stock supplemented with 8 μg/ml polybren and cells were centrifuged for 75 min at 1000 g and 32 °C. Viral stock was replaced with initial medium and cells were further cultured for four days. Recombinant IL-2 was added at day 3 (from initiating cell cultures) to a final concentration of 10 ng/ml. At day 5, differentiated Th1 and Th2 cells were isolated by Ficoll gradient and rested overnight in culture medium without cytokines or antibodies. On the next day cells were stimulated with 10 μg/ml of anti-CD3 and 2 μg/ml of anti-CD28 antibodies and successfully transformed cells were isolated by Fluorescence Activated Cell Sorting (FACS) based on their expression of GFP. Efficiency of retroviral transduction was in the range from 30 to 90% of live cells.

### Flow cytometry analysis and sorting

Flow cytometry analysis was performed using BD LSRFortessa and BD Canto analyzers (Becton Dickinson, Franklin Lakes, NJ) and antibodies listed in Supplementary Table S3. An average of 20,000 cells was used per sample. Data collection was performed by BD FACSDiva software. FlowJo software was used for data analysis. Cell sorting was performed using BD Influx sorter operated under BD FACS^TM^ software. An average of 10^6^ GFP^+^ cells was sorted per sample. Propidium Iodide and Fixable Viability Dye eFluor® 780 (eBioscience, San Diego, CA) were used for exclusion of dead cells.

### Cell fractionation and Western blotting

Cells were washed with ice-cold PBS, centrifuged 5 min at 500 g, resuspended in lysis buffer 1 (L1) (10 mM TrisHCl, pH 7.4, 10 mM NaCl, 3 mM MgCl2, 0.5% Nonidet P-40, 0.15 mM spermine and 0.5 mM spermidine) supplemented with protease inhibitors (Complete Inhibitor Cocktail, Roche Diagnostics Deutschland GmbH, Mannheim, Germany) and phosphatase inhibitors (Sigma Phosphatase Inhibitor Coctails ##2 and 3, Sigma-Aldrich, St. Louis, MO) and incubated on ice for 5 min. Nuclei were sedimented by centrifugation 5 min at 500 g and supernatant representing combined cytoplasmic and cell membrane fractions was mixed with 6x Laemmli buffer. Nuclei were washed 2 times in L1 buffer, resuspended in L1 buffer and supplemented with 6x Laemmli buffer. Total cell lysates were produced by resuspension of the cells in L1 buffer supplemented with 6x Laemmli buffer. Nuclear and total cell lysate samples were then treated for 1 min in cup-horn type ultrasound sonicator. All samples were then subjected to SDS-PAGE using 10% gels under reducing conditions and analyzed by Western blotting. All used antibodies are listed in Supplementary Table S3.

### ELISA

Analysis of IL-2 concentration by ELISA kit (eBioscience) was performed according manufacturer’s instruction.

### Quantitative mRNA analysis

RNA was isolated from cells using Tri-Reagent (Sigma-Aldrich), treated with RNase-free DNase I (Thermo Scientific, Waltham, MA) and converted to cDNA using RevertAid H Minus Reverse Transcriptase (Thermo Scientific) and random nonamer primers (Life Technologies, Grand Island, NY). Q-PCR was performed in StepOne Plus (Applied Biosystems, Foster City, CA, USA) Real-Time PCR system using Maxima SYBR Green/ROX qPCR Master Mix (Thermo Scientific). All primers used in this work are listed in Supplementary Table S4. All mRNA data were normalized to β-actin.

### Densitometry and statistic analysis

Initial processing of Western blot images (image rotation, assembly of sample panels) was performed using Adobe Photoshop CS4 Version 11.0 (Adobe Systems, San Jose, CA) and densitometry analysis was performed by ImageJ 1.42q freeware (http://rsb.info.nih.gov/ij). MS Excel 2010 (Microsoft Corp., Redmond, WA) was used for the statistical analysis and generation of graphs and histograms. Online statistical calculator was used for Mann Whitney U-test: http://scistatcalc.blogspot.de/2013/10/mann-whitney-u-test-calculator.html.

## Electronic supplementary material

Supplementary Information
