# Supplementary material for: Low level of Lck kinase in Th2 cells limits expression of CD4 co-receptor and S73 phosphorylation of transcription factor c-Jun
Source: Sci Rep. 2017 May 24;7:2339. doi: 10.1038/s41598-017-02553-y (PMC5443812; doi:10.1038/s41598-017-02553-y)
Supplement: Supplementary file 1 — Supplementary Information [file 41598_2017_2553_MOESM1_ESM.pdf]

Low level of Lck kinase in Th2 cells limits expression of CD4 co-receptor and S73 phosphorylation of transcription factor c-Jun

Yury V. Shebzukhov, Silke Stanislawiak, Taisiya R. Bezhaeva,

Sergei A. Nedospasov and Dmitry V. Kuprash.

Supplementary Information

Supplementary Table S1. Mean PerCP fluorescence of GFP$^+$ cells stained with anti-CD4 PerCP-conjugated antibodies

	Experiment 1	Experiment 2	Experiment 3	Experiment 4
**Th1-GFP**	13840	2344	4723	2632
**Th2-GFP**	10828	1222	3746	1162
**Th2-Lck**	14940	1610	6507	1906
**Th2-Fyn**	10603	1247	3533	1228
**Th2-CD4**			3794	1480

Naive CD4$^+$ cells were transduced with control (pMSCV-IRES-GFP), Lck (pMSCV-LCK-IRES-GFP), Fyn (pMSCV-FYN-IRES-GFP) or CD4 (pMSCV-CD4-IRES-GFP) encoding retroviruses and polarized under Th2 or Th1 (control vector only) conditions. 5 days after initiation of cell cultures GFP$^+$ cells were isolated and analyzed by flow cytometry. See Supplementary Figure S7B for graphical representation of the data normalized to Th2 cells transduced with control retrovirus.

Supplementary Table S2. Densitometry analysis of relative c-Jun S73 phosphorylation

	**Th2-GFP**			**Th2-Fyn**			**Th2-Lck**		
	**Control**	**5 min**	**15 min**	**Control**	**5 min**	**15 min**	**Control**	**5 min**	**15 min**
Experiment 1	0.12	0.79	1.34	0.34	0.92	1.75	0.46	1.21	2.07
Experiment 2	0.03	0.77	1.20	0.11	0.60	1.55	0.28	1.44	3.04
Experiment 3	0.17	0.40	0.91	0.52	0.91	1.48	0.33	0.90	3.39
**Average**	**0.11**	**0.66**	**1.15**	**0.32**	**0.81**	**1.59**	**0.36**	**1.18**	**2.83**
SD	0.07	0.22	0.22	0.20	0.18	0.14	0.10	0.27	0.68

Naive $CD4^+$ cells were transduced with control (pMSCV-IRES-GFP), Lck (pMSCV-LCK-IRES-GFP) and Fyn (pMSCV-FYN-IRES-GFP) encoding retroviruses and polarized under Th2 conditions. 5 days after initiation of cell culture GFP^+ cells were isolated, rested overnight without APCs, antibodies and cytokines and re-stimulated with soluble anti-CD3 (10 μg/ml) and anti-CD28 (2 μg/ml) antibodies. Western blot of nuclear fractions was performed using antibodies recognizing S73-phosphorylated and total transcription factor c-Jun. Signals obtained from pS73 c-Jun blots were normalized to corresponding signals from total c-Jun blots and further normalized to the mean signals ratio for each experiment.

Supplementary Table S3. List of antibodies

Antigen	Host	Clone and Isotype	Application (conjugate)	Supplier	Cat. #
β-Actin	Rabbit		WB	Santa Cruz	sc-130656
CD3ε	Arm. hamster	145-2C11, IgG1 κ	T cell polarization	BD Pharmingen	553057
CD3ε	Arm. hamster	145-2C11, IgG1 κ	T cell stimulation	DRFZ	
CD3-ζ	Mouse	6B10.2, IgG_1	WB	Santa Cruz	sc-1239
pY142 CD3-ζ	Rabbit	EP265(2)Y, IgG	WB	Epitomics	2280
CD4	Rat	GK1.5, IgG2b, κ	MACS (FITC), FC (PE or PerCP)	DRFZ	
CD4	Mouse	4SM95, IgG1 κ	WB	eBioscience	14-9766-80
CD25	Rat	pC61.5 IgG1 λ	MACS (Cy5)	DRFZ	
CD28	Syrian hamster	37.51, IgG2, λ1	T cell polarization	BD Pharmingen	553294
CD28	Syrian hamster	37.51, IgG2, λ1	T cell stimulation	DRFZ	
CD44	Rat	IM7, IgG2b	MACS/FC (PE)	DRFZ	
pT202, pY204 ERK	Rabbit	D13.14.4E	WB	Cell Signaling	4370
ERK	Mouse	L34F12, IgG1	WB	Cell Signaling	4696
Fyn	Mouse	FYN-59, IgG	WB	BioLegend	626502
IFNγ	Rat	AN 18.17.24	T cell polarization	DRFZ	
IFNγ	Rat	XMG1	FC (Cy5)	DRFZ	
IL4	Rat	11B11, IgG1	T cell polarization	DRFZ	
IL4	Rat	11B11, IgG1	FC (PE)	eBioscience	12-7041-41
IL12	Rat	C17.8, IgG2a	T cell polarization	DRFZ	
pT183, pY185 JNK	Rabbit	polyclonal	WB	Cell Signaling	9251
JNK	Rabbit	56G8, IgG	WB	Cell Signaling	9258
c-Jun	Rabbit	60A8, IgG	WB	Cell Signaling	9165
pS63 c-Jun	Rabbit	Y172, IgG	WB	Epitomics	1527
pS73 c-Jun	Rabbit	D47G9, IgG	WB, FC	Cell Signaling	3270
Lamin B	Goat	polyclonal	WB	Santa Cruz	sc-6217
Lck	Mouse	3A5, IgG_{2b}	WB	Santa Cruz	sc-433
Lck	Mouse	MOL 171, IgG1 κ	FC (Alexa Fluor 647)	BD Biosciences	558505
pY420 Fyn/ pY394 Lck	Rabbit	polyclonal	WB	Santa Cruz	sc-101728
NFATc2	Rabbit	polyclonal	WB	ImmunoGlobe	0112-02
pY783 PLCγ	Rabbit	EP2610Y, IgG	WB	Epitomics	2350
RelA	Rabbit	polyclonal	WB	Santa Cruz	sc-372
VAV1	Rabbit	IgG	WB	Enogene	E021165-1
pY174 VAV1	Rabbit	EP510Y	WB	Epitomics	2133
Goat IgG	Donkey	IgG, polyclonal	WB (HRP)	Bethyl	A50-101P
Rabbit IgG	Goat	IgG, polyclonal	WB (HRP)	Zymed	81-6120
Rabbit IgG	Goat	IgG, polyclonal	FC (AlexaFluor 647)	Thermo Scientific	A21246
Mouse IgG	Goat	IgG, polyclonal	WB (HRP)	Sigma-Aldrich	A8924
Isotype control	Rat	RTK4530, IgG2b κ	FC (PerCP)	Biolegend	400629
Isotype control	Rat	RTK2758, IgG2a κ	FC (PE)	Biolegend	400507
Isotype control	Mouse	MOPC-21, IgG1 κ	FC (Alexa Fluor 647)	Biolegend	400135

FC – Flow cytometry, WB – Western blot, MACS - magnetic-activated cell sorting

Supplementary Table S4. List of oligonucleotide primers

Amplicon	Forward primer	Reverse primer
Q-RT-PCR primers		
β-Actin	CTCCTgAgCgCAAgTACTCTgTg	TAAAACgCAgCTCAgTAACAgTCC
CD4	CACCTgTgCAAgAAgCAgAg	CAAgCgCCTAAgAgAgATgg
Lck_TV1	TgACgATCTCggggATCATgg	CTTgCTgTCCAgTgggACTATgg
Lck_TV2	gCTCCTTCAggCCTCTCTACATTC	CTTgCTgTCCAgTgggACTATgg
Lck_TV3	gTgAATAggCCAgAAggCTCCC	CTTgCTgTCCAgTgggACTATgg
IL2	AACTCCCCAggATgCTCAC	CgCAgAggTCCAAgTTCATC
IL4	TTgAACgAggTCACAggAgA	AAATATgCgAAgCACCTTgg
IL10	AgCTgAAgACCCTCAggATg	TggCCTTgTAgACACCTTgg
TNF	CAgCCTCTTCTCATTCCTgC	ggTCTgggCCATAgAACTgA
Cloning primers		
CD4	AATAgATCTATgTgCCgAgCCATCTCTCT	CTACTCgAgTCAgATgAgATTATggCTCTTCTg

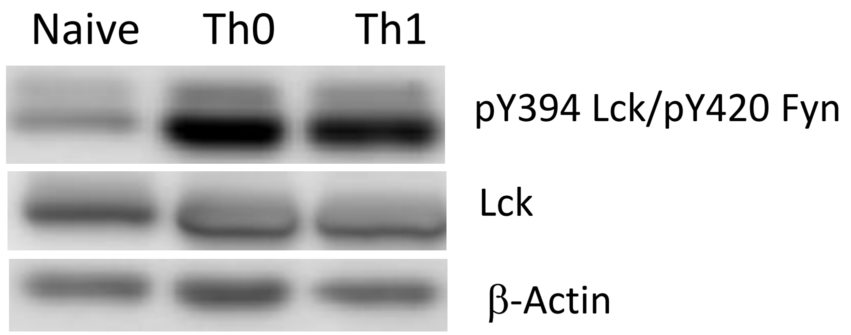

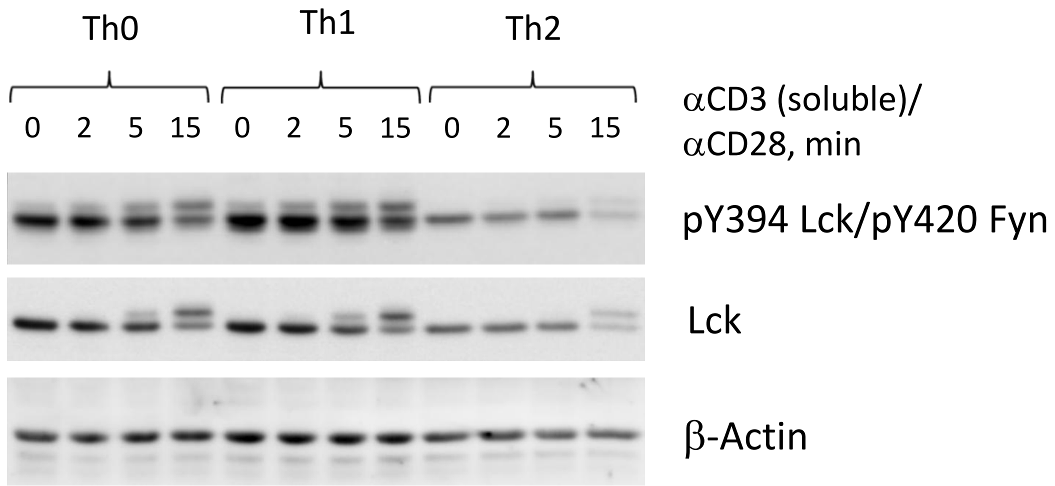

Supplementary Figure S1. Lck protein level in naïve and differentiated CD4$^+$ T cells
Naive CD4$^+$ T cells were polarized under neutral (Th0), Th1 and Th2 conditions for 5 days, rested overnight without APCs, antibodies and cytokines and re-stimulated with soluble anti-CD3 (10 µg/ml) and anti-CD28 (2 µg/ml) antibodies. Western blotting analysis of cytoplasmic /cell membrane fraction. Results of a representative experiments (of four experiments) are shown.

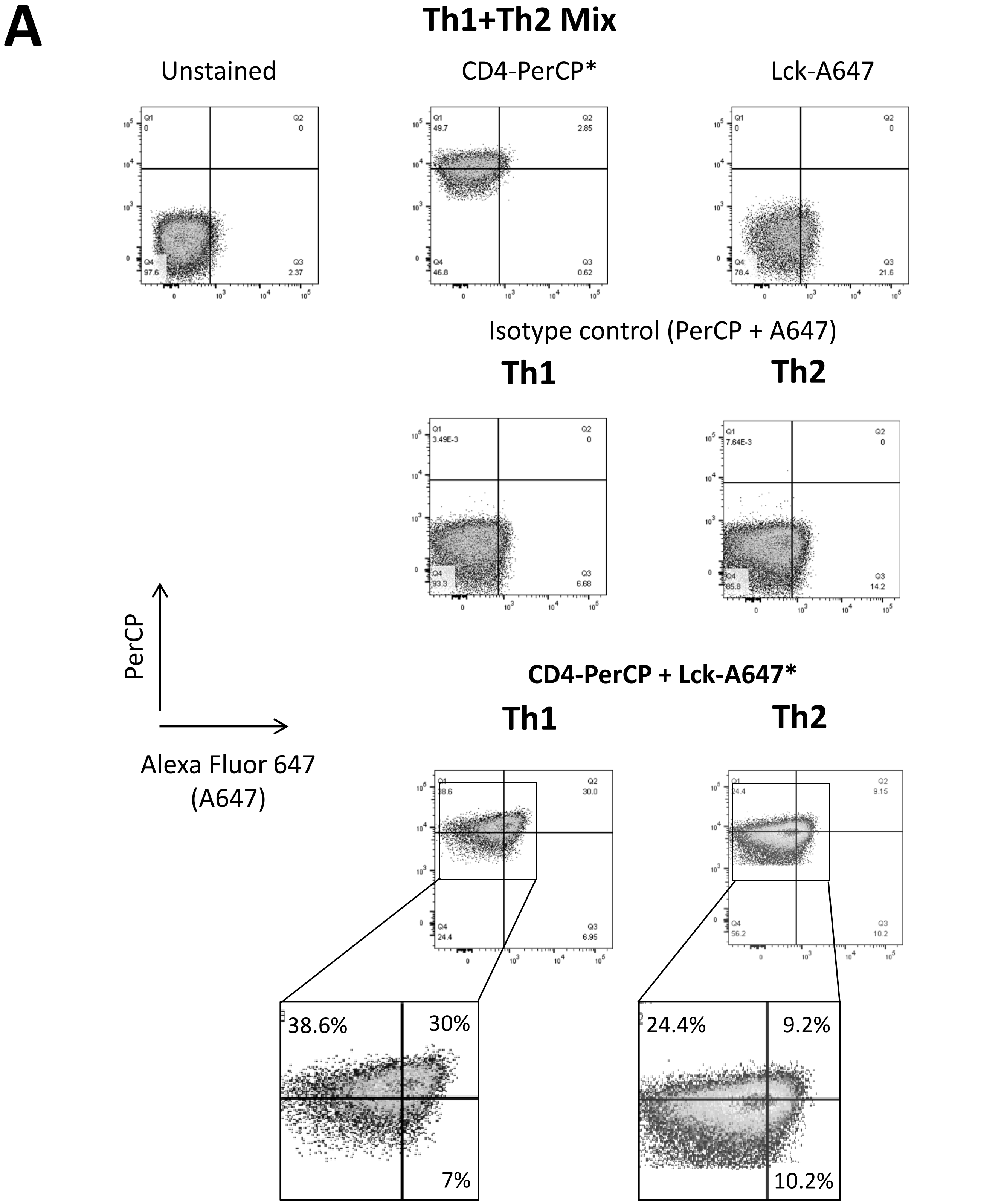

Supplementary Figure S2A. Correlation between Lck and CD4 expression
Naive $CD4^+$ cells were polarized under Th1 or Th2 conditions. 5 days after initiation of cell cultures cells were stained with anti-Lck or isotype control Alexa Fluor 647-labeled and anti-CD4 or isotype control PerCP-labeled antibodies and analyzed by flow cytometry. Results of a representative experiment of two experiments are shown. * Gated on $CD4^+$ cells

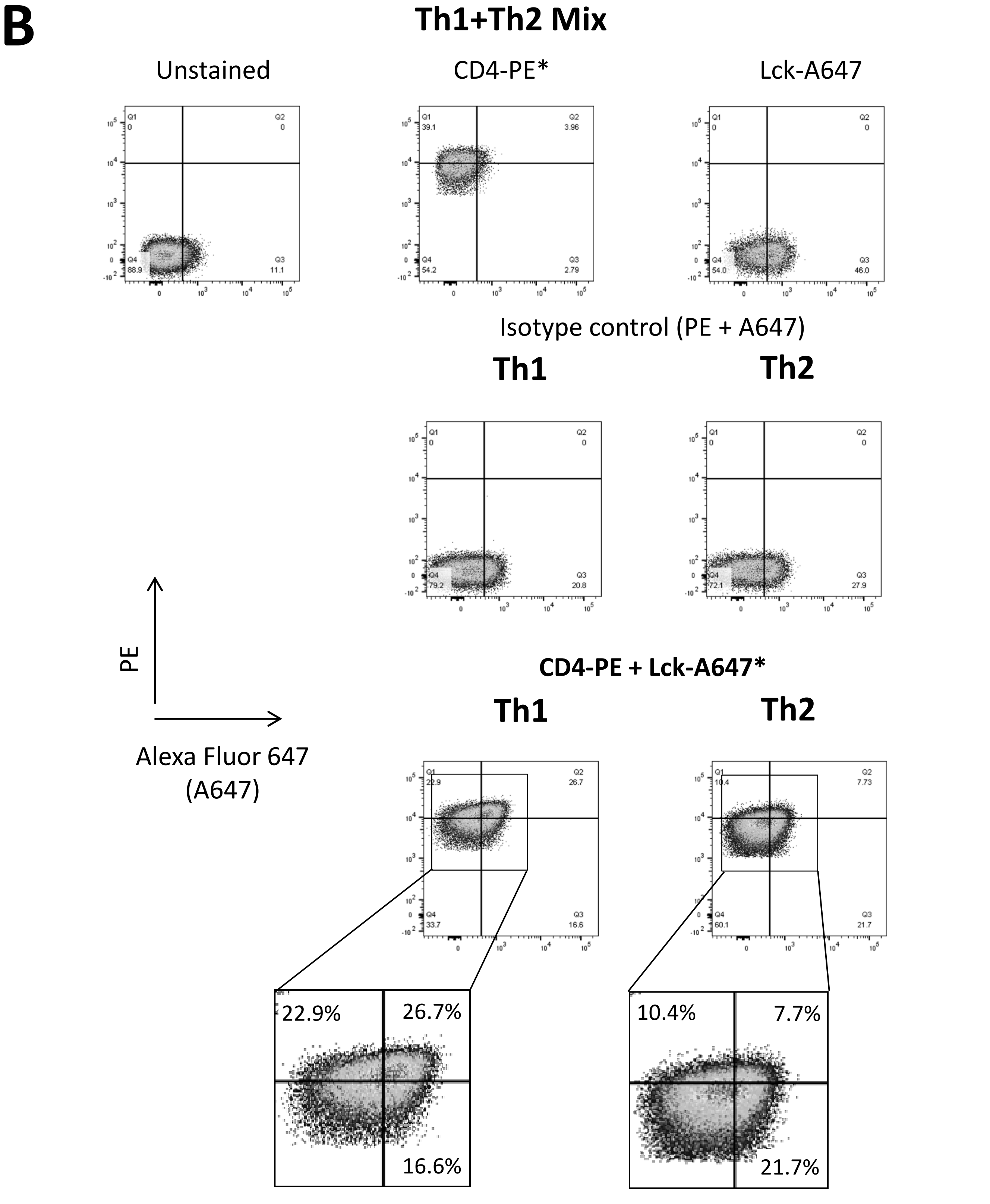

Supplementary Figure S2B. Correlation between Lck and CD4 expression
Naive CD4+ cells were polarized under Th1 or Th2 conditions. 5 days after initiation of cell cultures cells were stained with anti-Lck or isotype control Alexa Fluor 647-labeled and anti-CD4 or isotype control PE-labeled antibodies and analyzed by flow cytometry. Results of a representative experiment of two experiments are shown. * Gated on CD4+ cells

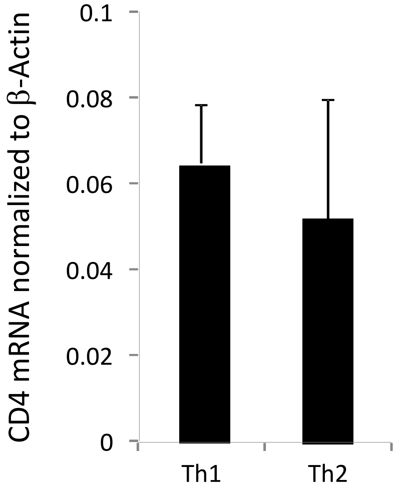

Supplementary Figure S3. Same level of CD4 mRNA in Th1 and Th2 cells
Naive $CD4^+$ T cells were polarized under Th1 and Th2 conditions for 5 days. CD4 mRNA expression in Th1 and Th2 cells was analyzed by reverse transcription and quantitative PCR. Average and standard deviation of seven independent experiments are shown.

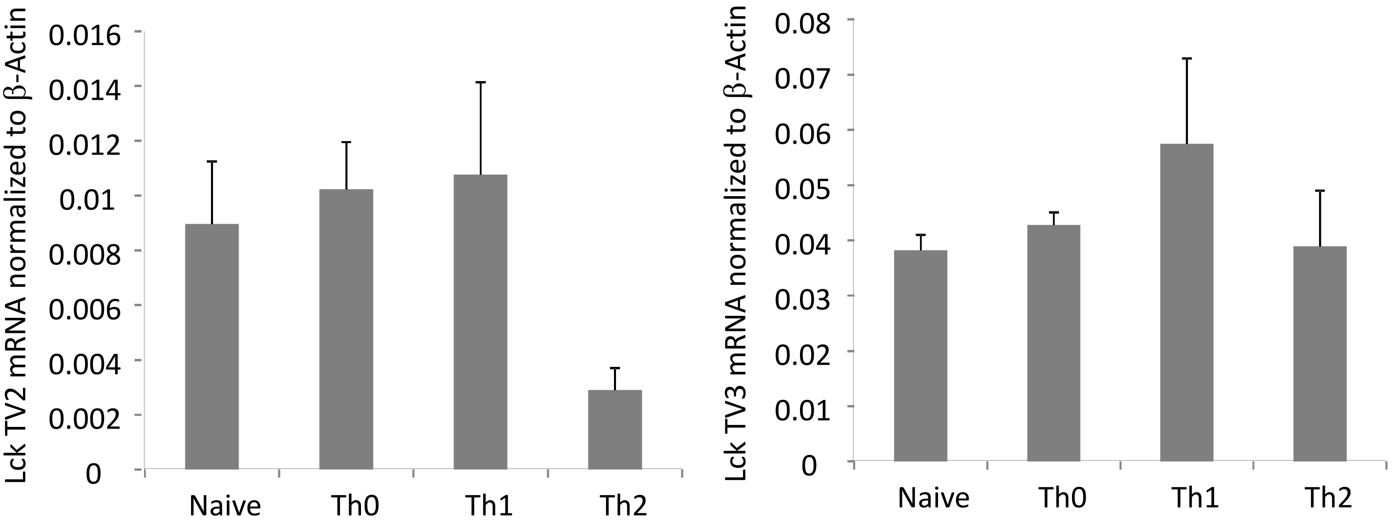

Supplementary Figure S4. Lck mRNA in naïve and differentiated $CD4^+$ T cells
Naive $CD4^+$ T cells were polarized under neutral (Th0), Th1 and Th2 conditions for 5 days. Lck mRNA expression in naïve and differentiated $CD4^+$ T cells was analyzed by reverse transcription and quantitative PCR. Lck TV2 – transcript from the proximal promoter, Lck TV3 – transcript from the distal promoter. Average and standard deviation of two independent experiments are shown.

A

Th1/Th2 ratio		GEO DataSet			
		GSE14308	GSE22081	GSE28929	**Average**
Affymetrix ID	1439146_s_at	2.06	2.31	1.25	**1.87**
	1439145_at	2.02	1.49	1.57	**1.69**
	1425396_a_at	1.70	1.22	1.17	**1.36**
	1457917_at	1.94	0.31	1.91	**1.38**
	**Average**	**1.93**	**1.33**	**1.47**	**1.58**

B

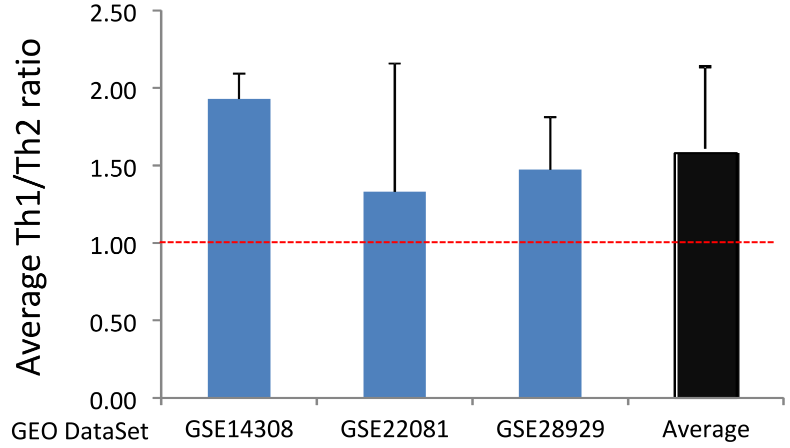

C

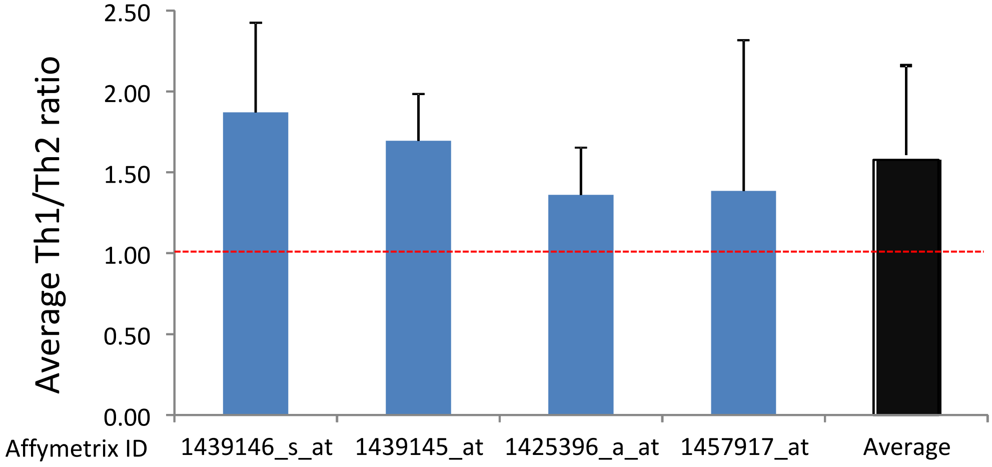

Supplementary Figure S5. Reduced Lck mRNA expression in mouse Th2 cells
Analysis of independent microarray DataSets deposited to Gene Expression Omnibus (GEO) repository (http://www.ncbi.nlm.nih.gov/geo/). A) Ratio of Th1 and Th2 signals . B) Average Th1/Th2 ratio for LCK-related Affymetrix IDs. C) Average Th1/Th2 ratio for DataSets GSE14308[1], GSE22081[2] and GSE28292[3] B,C) Average and standard deviation are shown.

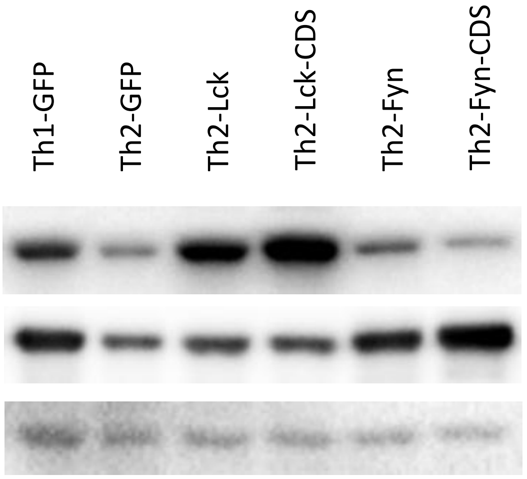

Supplementary Figure S6 (complete version of Figure 2A). Lck and Fyn expression in transduced Th2 cells

Naive $CD4^+$ cells were transduced with control (pMSCV-IRES-GFP), Lck (pMSCV-LCK-IRES-GFP and pMSCV-LCK-CDS-IRES-GFP) or Fyn (pMSCV-FYN-IRES-GFP and pMSCV-FYN-CDS-IRES-GFP) encoding retroviruses and polarized under Th2 or Th1 (control vector only) conditions. 5 days after initiation of cell cultures GFP^+ cells were isolated and analyzed by western blot. Staining was performed using the same blots as in Supplementary Figure S9. Correspondingly, the same β-Actin staining is shown there as loading control.

Results of a representative experiment of two experiments are shown.

pMSCV-LCK-IRES-GFP (Lck) and pMSCV-FYN-IRES-GFP (Fyn) vectors contain complete Lck (Ref. Seq. BC011474) and Fyn (Ref. Seq. BC032149) encoded clones including 3' and 5' untranslated sequences. Th2 cells transduced with corresponding retroviruses expressed Lck and Fyn at the levels comparable with ones in control Th1 cells.

pMSCV-LCK-CDS-IRES-GFP (Lck-CDS) and pMSCV-FYN-CDS-IRES-GFP (Fyn-CDS) vectors contain only Lck and Fyn coding sequences. Since Th2 cells transduced with Lck-CDS and Fyn-CDS retroviruses expressed Lck and Fyn, correspondingly, at the levels higher than the ones in control Th1 cells, these vectors were not used in further experiments unless otherwise specified.

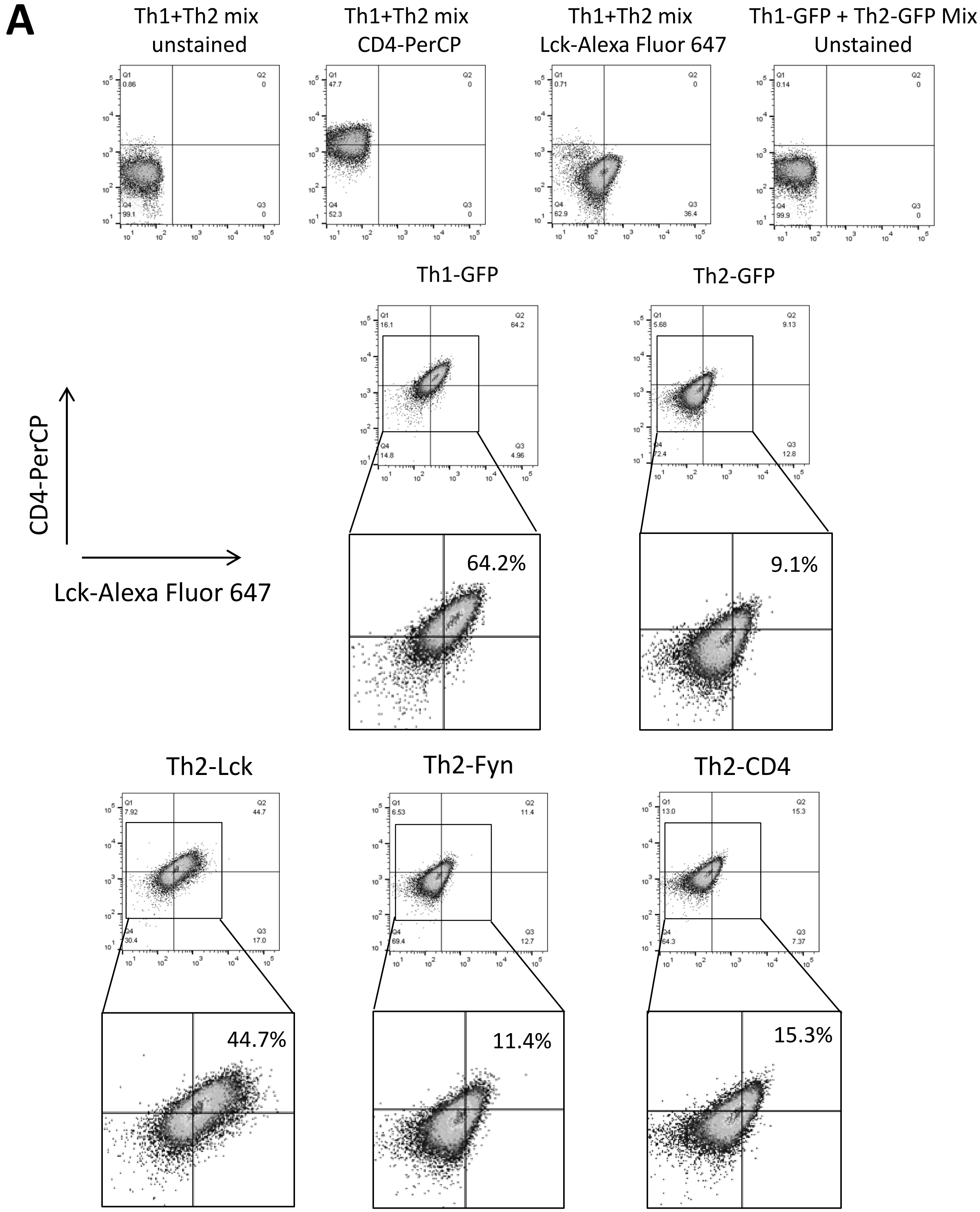

Supplementary Figure S7A. Lck increases surface CD4 expression in Th2 cells

A) Naive CD4$^+$ cells were transduced with control (pMSCV-IRES-GFP), Lck (pMSCV-LCK-IRES-GFP), Fyn (pMSCV-FYN-IRES-GFP) or CD4 (pMSCV-CD4-IRES-GFP) encoding retroviruses and polarized under Th2 or Th1 (control vector only) conditions. 5 days after initiation of cell cultures GFP$^+$ cells were isolated and analyzed by flow cytometry. Results of a representative experiment of four experiments are shown.

B

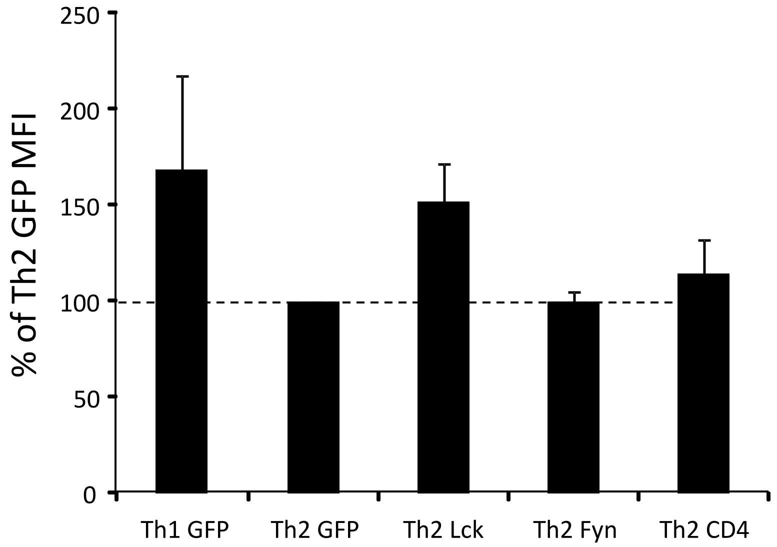

C

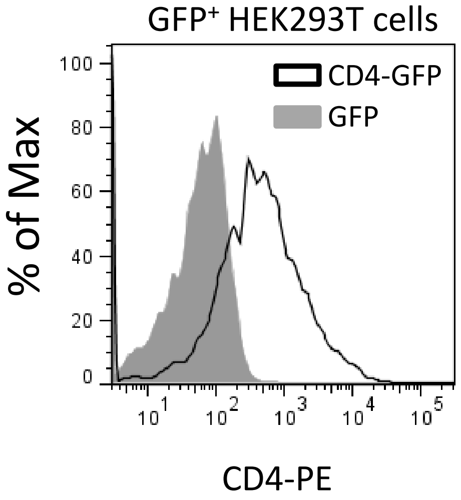

D

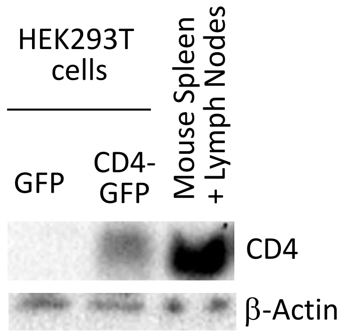

Supplementary Figure S7B-D. Lck increases surface CD4 expression in Th2 cells
B) Mean PerCP fluorescence of GFP$^+$ cells shown in Suppl. Fig. S7A was normalized to that of Th2 cells transduced with control retrovirus. Average and standard deviation of four experiments are shown for all experimental groups with the exception of CD4-transduced Th2 cells, since that group was included only in two experiments. See Suppl. Table S1 for raw data used to generate the histogram. C, D) The functionality of the expression vectors was tested in HEK293T cells transfected by control (pMSCV-IRES-GFP) or CD4 (pMSCV-CD4-IRES-GFP) encoding vectors. CD4 expression was assessed by flow cytometry (C) and by western blot of isolated GFP$^+$ cells (D).

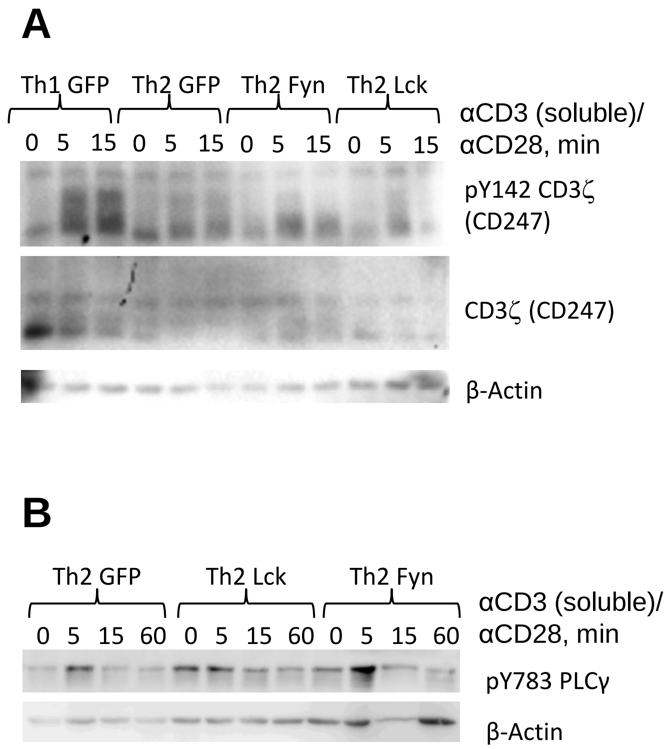

Supplementary Figure S8. TCR-mediated signaling pathways are unaffected by Lck and Fyn overexpression in Th2 cells

Naive CD4$^+$ cells were transduced with control (pMSCV-IRES-GFP), Lck (pMSCV-LCK-IRES-GFP) or Fyn (pMSCV-FYN-IRES-GFP) encoding retroviruses and polarized under Th1 or Th2 conditions. 5 days after initiation of cell cultures GFP$^+$ cells were isolated, rested overnight without APCs, antibodies and cytokines and re-stimulated with anti-CD3 (10 µg/ml) and anti-CD28 (2 µg/ml) antibodies. Cytoplasmic /cell membrane fraction was analyzed by western blot. In panel A**,** staining was performed using the same blots as in the upper panel of Suppl. Fig. S10A and in the Suppl. Fig. S11A. Correspondingly, the same β-Actin staining is shown as loading control. Results representative of two independent experiments are shown.

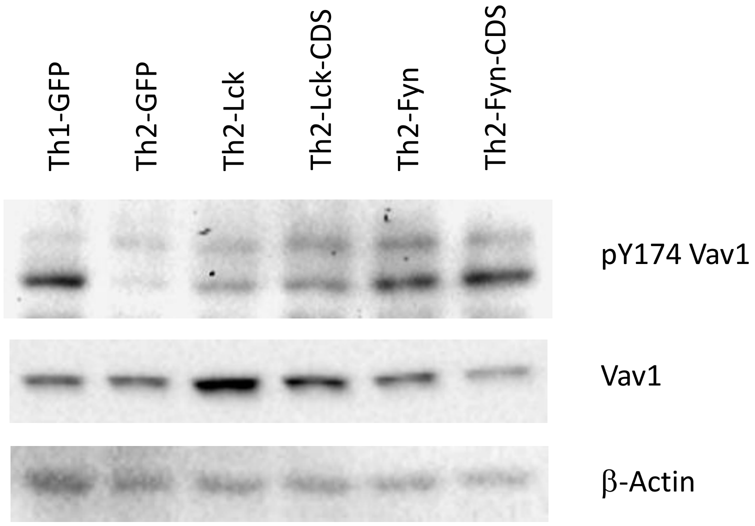

Supplementary Figure S9 (complete version of Figure 3A). Overexpression of Fyn increases pY174 phosphorylation of guanine nucleotide exchange factor Vav1

Naive CD4^{+} cells were transduced with control (pMSCV-IRES-GFP), Lck (pMSCV-LCK-IRES-GFP and pMSCV-LCK-CDS-IRES-GFP) or Fyn (pMSCV-FYN-IRES-GFP and pMSCV-FYN-CDS-IRES-GFP) encoding retroviruses and polarized under Th2 or Th1 (control vector only) conditions. 5 days after initiation of cell cultures GFP^{+} cells were isolated and analyzed by western blot. Staining was performed using the same blots as in Supplementary Figure S9. Correspondingly, the same β-Actin staining is shown there as loading control.

Results of a representative experiment of two experiments are shown.

pMSCV-LCK-IRES-GFP (Lck) and pMSCV-FYN-IRES-GFP (Fyn) vectors contain complete Lck (Ref. Seq. BC011474) and Fyn (Ref. Seq. BC032149) encoded clones including 3' and 5' untranslated sequences. Th2 cells transduced with corresponding retroviruses expressed Lck and Fyn at the levels comparable with ones in control Th1 cells.

pMSCV-LCK-CDS-IRES-GFP (Lck-CDS) and pMSCV-FYN-CDS-IRES-GFP (Fyn-CDS) vectors contain only Lck and Fyn coding sequences. Since Th2 cells transduced with Lck-CDS and Fyn-CDS retroviruses expressed Lck and Fyn, correspondingly, at the levels higher than the ones in control Th1 cells (Suppl. Fig. S6), these vectors were not used in further experiments unless otherwise specified.

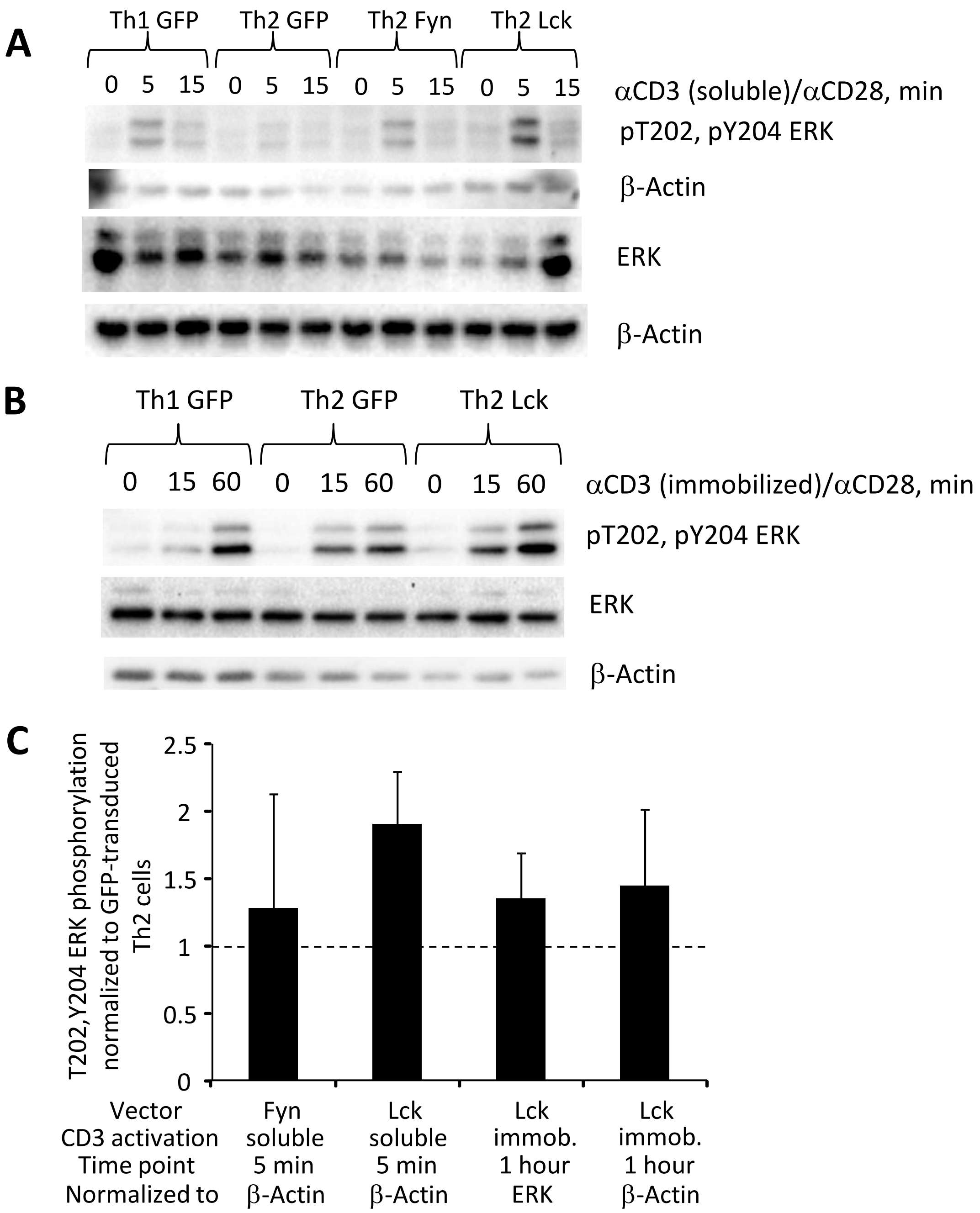

Supplementary Figure S10. ERK pathway activation is moderately augmented by Lck overexpression in Th2 cells

Naive CD4$^+$ cells were transduced and polarized as described in legend to Suppl. Fig. S7. 5 days after initiation of cell cultures GFP$^+$ cells were isolated, rested overnight without APCs, antibodies and cytokines and re-stimulated with anti-CD3 (10 µg/ml) (A – soluble, B – immobilized) and soluble anti-CD28 (2 µg/ml) antibodies. A) cytoplasmic /cell membrane fraction was analyzed by western blot. B) western blot of total cell lysates. In the upper panel of A**,** staining was performed using the same blots as in Supplementary Figures S8A and S11A. Correspondingly, the same β-Actin staining is shown there as loading control. Results representative of two independent experiments are shown.

C) Densitomenry analysis of T202,Y204 ERK phosphorylation at optimal time points (as defined in A and B). Everage value and standard deviation of two independent experiments are shown.

A

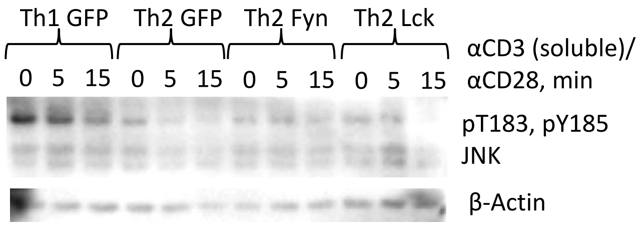

B

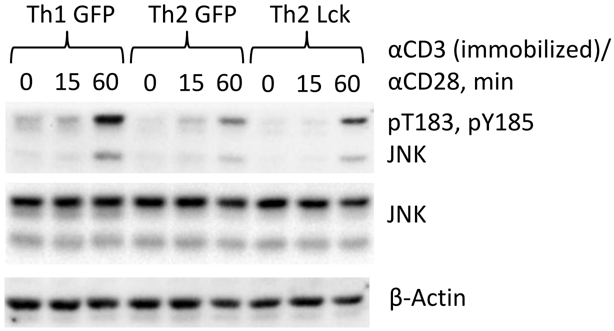

Supplementary Figure S11. JNK pathway activation is unaffected by Lck and Fyn overexpression in Th2 cells

Naive $CD4^+$ cells were transduced and polarized as described in legend to Suppl. Fig. S7. 5 days after initiation of cell cultures GFP^+ cells were isolated, rested overnight without APCs, antibodies and cytokines and re-stimulated with anti-CD3 (10 µg/ml) (A – soluble, B – immobilized) and soluble anti-CD28 (2 µg/ml) antibodies. A: cytoplasmic /cell membrane fraction was analyzed by western blot. B: western blot of total cell lysates. In panel A, staining was performed using the same blots as in Supplementary Fig. S8A and in two upper panels of Supplementary Fig. S10A. Correspondingly, the same image of β-Actin staining is shown as loading control. Results representative of two independent experiments are shown.

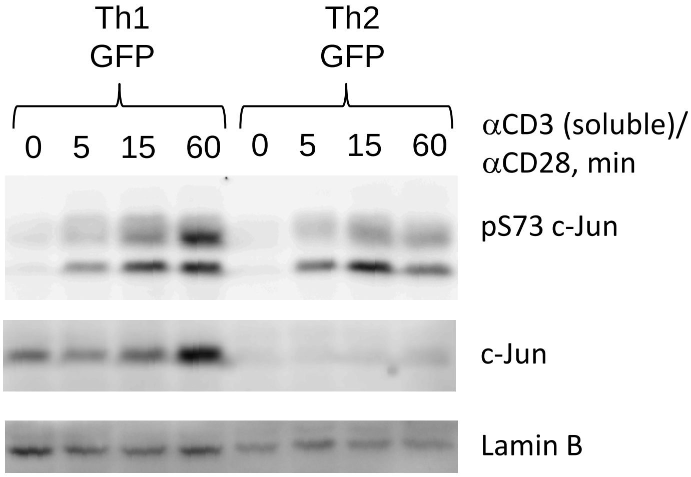

Supplementary Figure S12. pS73 c-Jun phosphorylation in Th1 and Th2 cells transduced with control vector

Naive CD4$^+$ cells were transduced with control pMSCV-IRES-GFP vector and polarized under Th1 and Th2 conditions. 5 days after initiation of cell culture GFP$^+$ cells were isolated, rested overnight without APCs, antibodies and cytokines and re-stimulated with soluble anti-CD3 (10 µg/ml) and anti-CD28 (2 µg/ml) antibodies. Western blot of nuclear fraction. Results of a representative experiment of three experiments are shown.

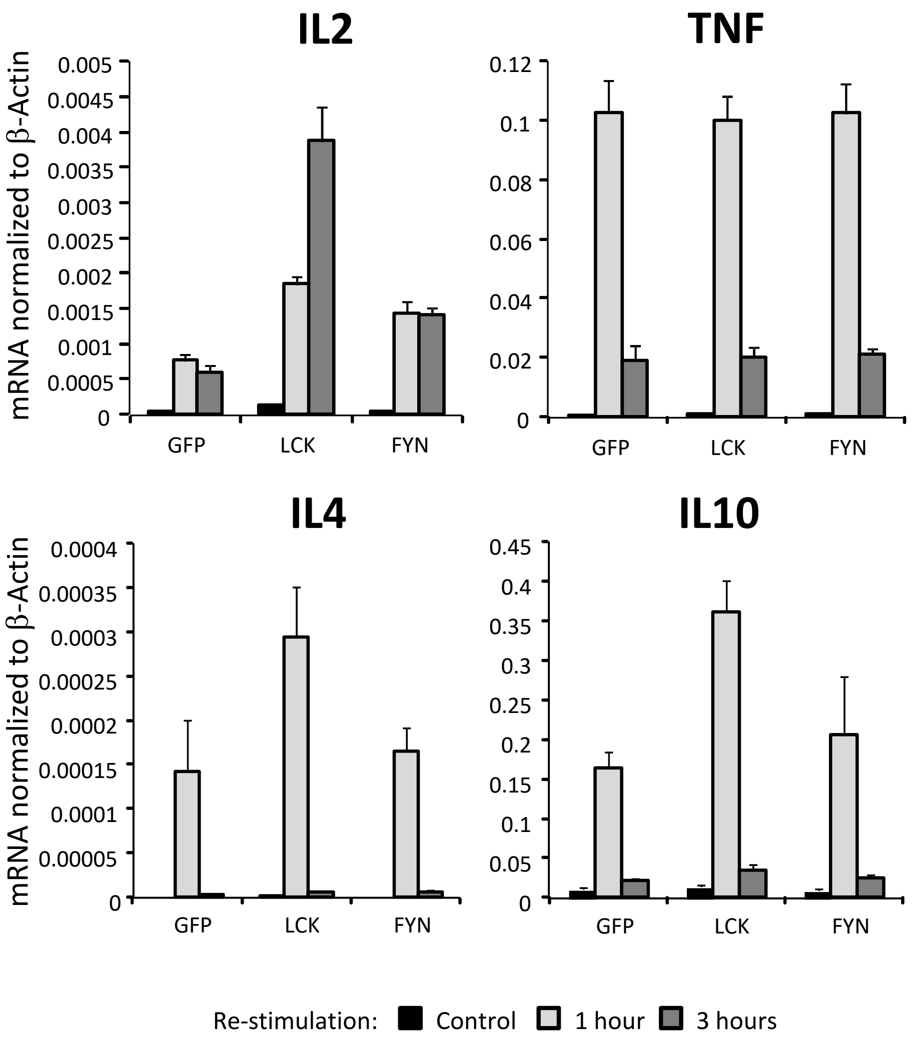

Supplementary Fig. S13. Ectopic overexpression of Lck in Th2 cells increases transcription of *IL2* gene

Naive CD4^{+} cells were transduced as described in the legend to Suppl. Fig. S7 and polarized under Th2 conditions. 5 days after initiation of cell cultures GFP^{+} cells were isolated, rested overnight without APCs, antibodies and cytokines and re-stimulated with soluble anti-CD3 (10 µg/ml) and anti-CD28 (2 µg/ml) antibodies. mRNA levels for the indicated cytokines were analyzed by reverse transcription and quantitative PCR.. Results of a representative experiment of four experiments are shown. A trend of moderate overexpression of IL4 and IL10 mRNAs in Lck-transduced cells did not reach statistical significance.

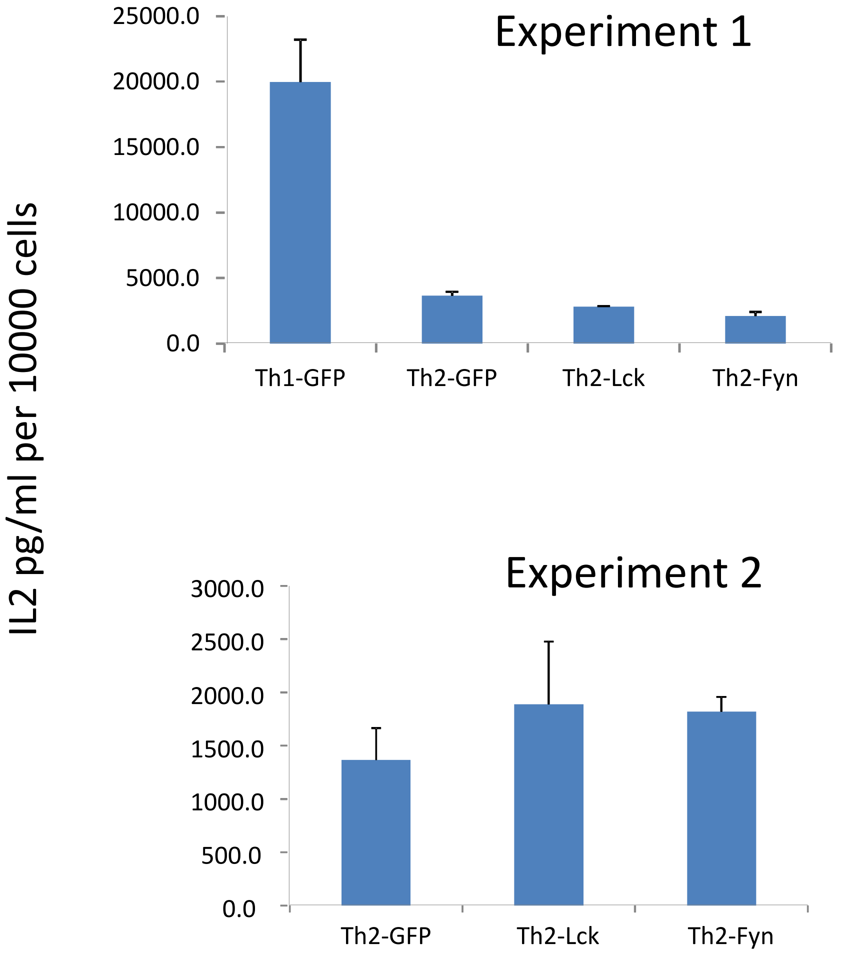

Supplementary Figure S14. IL2 protein secretion by Th2 cells overexpressing Lck and Fyn
Naive $CD4^+$ cells were transduced with control (pMSCV-IRES-GFP), Lck (pMSCV-LCK-IRES-GFP) or Fyn (pMSCV-FYN-IRES-GFP) encoding retroviruses and polarized under Th1 (control vector only) or Th2 conditions. 5 days after initiation of cell cultures GFP^+ cells were isolated, rested overnight without APCs, antibodies and cytokines and re-stimulated overnight with immobilized anti-CD3 (10 µg/ml) and soluble anti-CD28 (2 µg/ml) antibodies. IL2 protein concentration in supernatants is assessed by ELISA. Results of two of three independent experiments are shown.

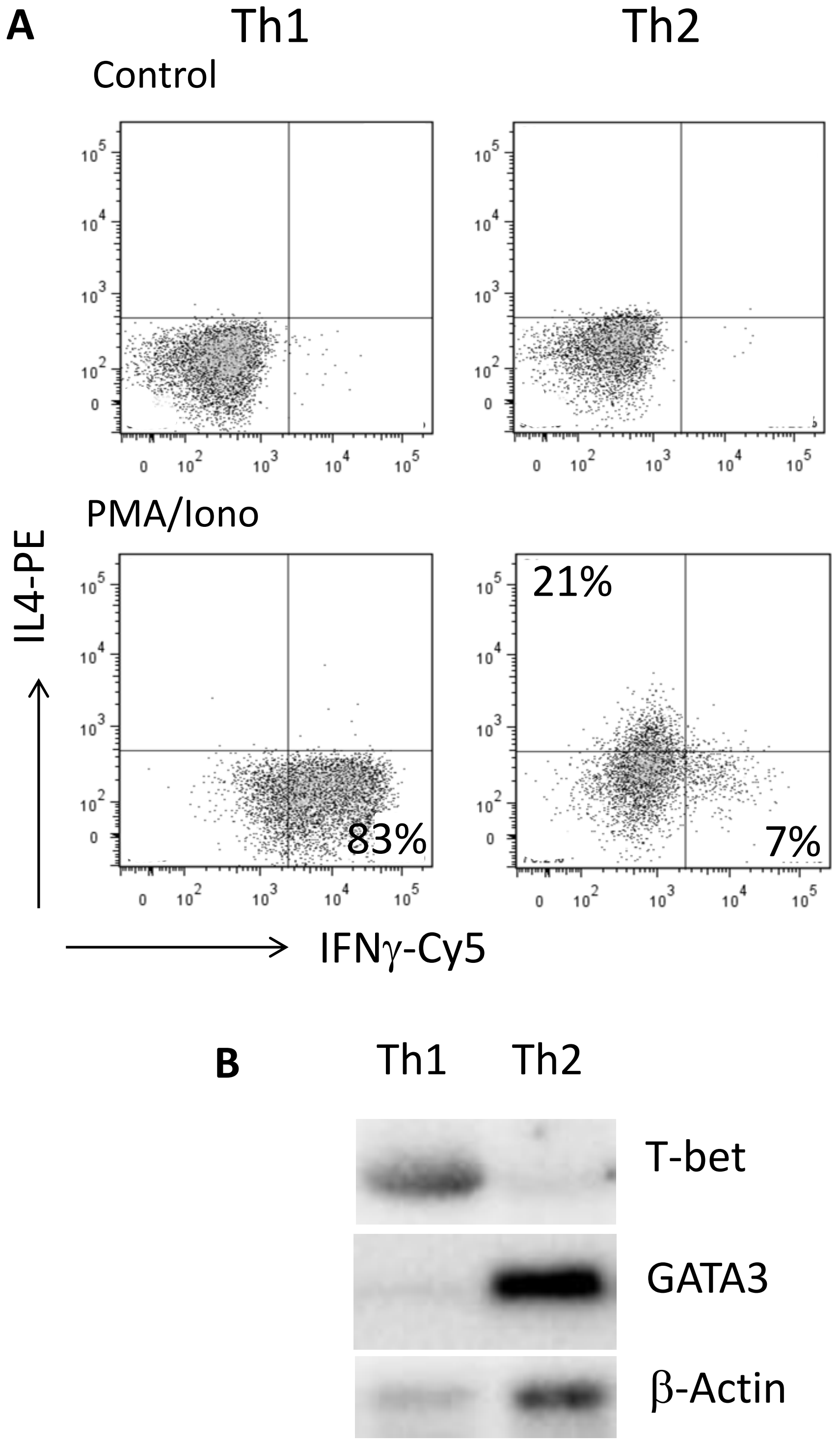

Supplementary Figure S15. Control of Th1 and Th2 polarization
Naive $CD4^+$ T cells were polarized under neutral Th1 and Th2 conditions for 5 days, rested overnight without APCs, antibodies and cytokines and re-stimulated with 10 ng/ml PMA and 1 μg/ml Ionomycin (PMA/Iono) for 5 hours. A) Flow cytometry analysis of IFNγ and IL4 expression in re-stimulated Th1 and Th2 cells. B) Western blotting analysis of T-bet and GATA3 expression in total cell lysates of resting Th1 and Th2 cells. A,B) Results of a representative experiment of four experiments are shown.

Supplementary references

1 Wei, G. *et al.* Global Mapping of H3K4me3 and H3K27me3 Reveals Specificity and Plasticity in Lineage Fate Determination of Differentiating CD4+ T Cells. *Immunity* **30**, 155-167, doi:10.1016/j.immuni.2008.12.009 (2009).

2 Wei, L. *et al.* Discrete Roles of STAT4 and STAT6 Transcription Factors in Tuning Epigenetic Modifications and Transcription during T Helper Cell Differentiation. *Immunity* **32**, 840-851, doi:10.1016/j.immuni.2010.06.003 (2010).

3 Horiuchi, S. *et al.* Genome-wide analysis reveals unique regulation of transcription of Th2-specific genes by GATA3. *J Immunol* **186**, 6378-6389, doi:10.4049/jimmunol.1100179 (2011).
